# The prevalence and clinical significance of EGFR mutations in non-small cell lung cancer patients in Egypt: a screening study

**DOI:** 10.1186/s43046-024-00251-1

**Published:** 2024-12-23

**Authors:** Asmaa A. Helal, Ibrahim H. Kamal, Ahmed Osman, Magdy Youssef, Adel K. Ibrahim

**Affiliations:** 1 Department of Biochemistry, Faculty of Science, Ain Shams University, Cairo, 11566 Egypt; 2 Biotechnology Program, Institute of Basic and Applied Sciences, Egypt-Japan University of Science and Technology, Alexandria, 21934 Egypt; 3 AstraZeneca International (UK Office), London, UK; 4 Department of Clinical Pathology, Faculty of Veterinary Medicine, Cairo University, Giza, 12211 Egypt

**Keywords:** Lung cancer, NSCLC, Mutation detection, EGFR mutations, Real-time PCR, 19 Del, L858R, T790M, Gender, Smoking, Age

## Abstract

**Background:**

Lung cancer is a form of cancer that is responsible for the largest incidence of deaths attributed to cancer worldwide. Non-small cell lung cancer (NSCLC) is the most prevalent of all the subtypes of the disease. Treatment with tyrosine kinase inhibitors (TKI) may help some people who have been diagnosed with non-small cell lung cancer. The presence of actionable mutations in the epidermal growth factor receptor (EGFR) gene is a key predictor of how a patient will respond to a TKI. Thus, the frequency of identification of mutations in EGFR gene in patients with NSCLC can facilitate personalized treatment.

**Objective:**

The objective of this study was to screen for mutations in the EGFR gene and to investigate whether there is a correlation between the screened mutations and various clinical and pathological factors, such as gender, smoking history, and age, in tissue samples from patients with NSCLC.

**Methods:**

The study comprised 333 NSCLC tissue samples from 230 males and 103 females with an average age of 50 years. Exons 18–21 of the EGFR gene have been examined using real-time PCR. Using SPSS, correlations between clinical and demographic variables were examined, and EGFR mutation and clinical features associations were studied.

**Results:**

The study’s findings revealed that the incidence rate of EGFR mutation was 24.32% (81/333), with partial deletion of exon 19 (19-Del) and a point mutation of L858R in exon 21 accounting for 66.67% (*P* < 0.001) and 28.40% (*P* < 0.001) of the mutant cases, respectively. Patients who had the T790M mutation represent 4.94% (*P* = 0.004) of total number of patients. Females harbored EGFR mutations (54.32%) with higher frequency than men (45.68%) (*P* < 0.001), while nonsmokers had EGFR mutations (70.37%) more frequently than current smokers (29.63%) (*P* < 0.001).

**Conclusion:**

The screening study conducted in Egypt reported that the EGFR mutations prevalence was 24.32% among Egyptians with NSCLC. The study also found a slight gender bias, with females having an incidence rate of these mutations higher than males. Additionally, nonsmokers had higher rates of mutations in EGFR gene compared to smokers. According to the findings, somatic EGFR mutations can be employed as a diagnostic tool for non-small cell lung cancer in Egypt, and they can be implemented in conjunction with clinical criteria to identify which patients are more likely to respond favorably to TKIs.

**Supplementary Information:**

The online version contains supplementary material available at 10.1186/s43046-024-00251-1.

## Background

The lung cancer incidence is an important global health issue, and it cause many cancer-related deaths compared any other type of cancer among both females and males, accounting for around 25% of all cancer-related deaths [1, 2]. The two major lung cancer subtypes are non-small cell lung cancer (NSCLC) and small cell lung cancer (SCLC). SCLC accounts for a smaller proportion of diagnosed cases, approximately 15%, while NSCLC is the more prevalent subtype, constituting around 85% of cases diagnosed with lung cancer [2, 3]. In accordance with the World Health Organization (WHO), lung cancer induces many deaths worldwide, with 1.76 million deaths per year attributed to it. Concurrently, incidence is growing with an estimate of 2.09 million new cases worldwide [4]. In Egypt, lung cancer is a significant health issue responsible for 5.7% of all cancer cases and 7.3% of all cancer-related deaths [5]. It is the most prevalent cancer form among males and the third prevalent cancer form among females. Additionally, the lung cancer incidence showed an increase in Egypt during the past few decades, with males being more likely to be affected than females [6]. In the Middle East, prevalence of lung cancer in the population varies by country, with rates ranging from 5.6 to 11.8 per 100,000 population [6, 7].

Lung cancer cells have many targetable genetic mutations in many proto-oncogenes, c-roes oncogene 1 (ROS1), epidermal growth factor receptor (EGFR), Kirsten rat sarcoma viral oncogene homolog (KRAS), and anaplastic lymphoma kinase (ALK) which are some of the driver genes that have been identified through recent advancements in molecular biology. These genes are important for the initiation of NSCLC as well as the disease’s progression [8, 9]. The transmembrane receptor tyrosine kinase, known as EGFR, has a crucial function in regulation of the cell differentiation, proliferation, and growth, and research have demonstrated that the EGFR signaling pathway is intimately linked to cancer cell proliferation and could act as a promising target for cancer treatment [10]. Abnormalities in the EGFR gene, such as mutations or overexpression, have been linked to an unfavorable prognosis in various cancers, including NSCLC [8]. Mutation identification is used widely to evaluate therapy approaches, and it has been determined that EGFR is a leading biomarker for determining how well a patient will respond to targeted therapy [11]. In 2004, a significant breakthrough was made when activating mutations in the EGFR gene were identified as the underlying cause of a positive response to treating via EGFR tyrosine kinase inhibitors (TKIs) in a subgroup of NSCLC patients [12, 13]. As a result of this finding, there was an uptick in research into potential treatments for aggressive tumors. In patients diagnosed with NSCLC, the examination of EGFR mutational status has become a crucial prerequisite [8].

The most common actionable alterations in NSCLC are EGFR mutations. “Actionable” mutations are those that can be addressed by certain medications or therapies [9]. The prevalence of EGFR mutations varies by population, with East Asians having the highest occurrence and Africans having the lowest [14]. On the other side, mutations in EGFR gene are still regarded as the most common actionable alterations in NSCLC across all demographics [15]. The therapy for NSCLC has been modified because of the identification of EGFR mutations. Patients who have EGFR mutations have a higher chance of responding favorably to targeted therapies as tyrosine kinase inhibitors than patients who do not have these mutations [8]. As a result, the EGFR mutational status has become an important consideration in identifying the best therapy strategy for NSCLC patients. Furthermore, the discovery of new molecular targets, such as ALK and ROS1, has increased the number of targeted medicines available to NSCLC patients [16, 17]. Many NSCLC patients’ prognoses and quality of life have improved because of the development of these targeted medicines. EGFR mutations that associate higher response to EGFR-TKIs (e.g., gefitinib, erlotinib, and afatinib) are more likely to be found in females and nonsmokers [8].

The EGFR protein was located on the cell surface and is activated by binding it to its ligand [18]. G719X mutations on exon 18, exon 19 partial deletions (19-Del), S768I mutation on exon 20, and L861Q and L858R mutations on exon 21 are sensitizing mutations. 19 deletions and L858R-sensitizing mutations account for more than 90% of triggering the EGFR gene mutations [11, 19], while resistance mutations, such as T790M mutation, C797S mutation, and three insertion mutations, are located on exon 20 [19, 20]. Regarding EGFR-sensitizing mutations, the majority involve L858R in exon 21 (about 40%) and exon 19 in-frame partial deletions (about 45%) [19]. These mutations increase EGFR kinase activity, which causes downstream signaling pathways to become hyperactive [21–23]. Uncontrolled activity of EGFR gene in case of NSCLC, which can be resulted from sensitizing mutations in the tyrosine kinase domain of EGFR, is a well-recognized oncogenic mechanism in NSCLC pathology. This mechanism is able to predict NSCLC susceptibility to TKIs, including erlotinib and gefitinib [23, 24]. The EGFR TKIs introduction was regarded as the turning point in lung cancer therapy [25, 26]. As a result of the discovery of EGFR-sensitizing mutations, various clinical guidelines now recommend the administration of TKIs as the primary treatment approach for NSCLC patients with these mutations, as well as for those who are at advanced or metastatic stages of NSCLC [27]. Patients having EGFR mutation-positive NSCLC typically have a more favorable prognosis compared to patients having EGFR mutation-negative NSCLC [28, 29]. Thus, testing for EGFR mutations is a vital component in the treatment-decision process [30]. More sensitive methods for identifying mutations have been developed given recent breakthroughs in molecular techniques [31]. Real-time quantitative polymerase chain reaction (qPCR) using certain probes and amplified refractory mutation system (ARMS) technology which is a PCR-bases method are known for their increased sensitive and specific potentialities in identifying frequent EGFR mutations in formalin-fixed paraffin-embedded (FFPE) tumor tissue, compared to the traditional method of direct sequencing [32]. While EGFR mutations are frequent in particular patient groups, such as female nonsmokers, they can occur in all types of NSCLC and in patients with different demographic and clinical characteristics. EGFR mutation detection is critical for guiding therapy options and improving patient outcomes [12, 13]. The study’s goal is to screen for the EGFR mutations frequency in Egyptian NSCLC patients and the frequency of mutation incidence with respect to clinical and demographic characteristics, comprising smoking history, gender, and age in the study population.

## Methods

### Study design

This study was conducted between 2019 and 2020, complying with all aspects of good clinical practice (GCP), including human rights, legal regulatory requirements, and AstraZeneca’s code of ethics. The test was performed on paraffin block remains following the histopathological testing (Cairo, Egypt) after the patient previous approval and consent signing according to AstraZeneca’s code of ethics. All enrolled patients had previously signed a written consent form to be included in the demographic and molecular studies which improved research values and ensured human rights policies that adhere to the code of ethics to deliver life-changing medicine. The study was carried out on the residual formalin-fixed paraffin-embedded tissue sections from newly diagnosed patients with NSCLC which were analyzed during the study period of total number of 333 formalin-fixed paraffin-embedded (FFPE) blocks. There were three main phases to the process: (1) extracting of the DNA, (2) real-time PCR amplification, and (3) analyzing the results.

### Patients data

The FFPE block samples were divided into 230 males (69.07%) and 103 females (30.93%), with a median age of 61 years and different smoking history. Most of the patients (163 patients, 48.95%) were in the age range of 61–80, followed by the age range from 41 to 60 (140 patients, 42.04%). The age range of 20–40 was constituted by 23 patients (6.91%). The range with lowest number of patients was the group more than 80 years (seven patients, 2.10%). The number of patients who smoke (153 patients, representing 45.95%) is relatively lower than the number of patients who do not smoke (180 patients, representing 54.05%) (Table 1).

**Table 1:** The study population demographic

**Parameters**		***N* = 333**	**Percentage**
**Age range (median)**	22–89 (median = 61)		
	20–40	23	6.91%
	41–60	140	42.04%
	61–80	163	48.95%
	> 80	7	2.10%
**Smoking status**	Smoker	153	45.95%
	Non-smoker	180	54.05%
**Gender**	Male	230	69.07%
	Female	103	30.93%

### DNA isolation

To extract DNA from formalin-fixed paraffin-embedded (FFPE) tissue blocks of solid tumor samples, sections of 5- to 10-μm-thick tumor underwent de-paraffinization using a series of xylene followed by ethanol washes as per the method described in Kokkat et al. [33]. DNA was extracted from the de-paraffinized tissue following the manufacturer’s instructions, using the QIAamp DNA FFPE Tissue kit (Qiagen, Hilden, Germany). The extracted DNA was then stored at 20 °C until it was required.

### Detection of EGFR mutations

For the detection of EGFR mutations, two kits were used in this study. The therascreen EGFR RGQ PCR Kit (Qiagen Manchester Ltd., Manchester, UK) was used to detect 29 significant somatic mutations in exons 18, 19, 20, and 21 in the EGFR gene, including the most prevalent mutations such as 19 deletions in exon 19, 3 insertions in exon 20, G719X in exon 18, S768I and T790M in exon 20, and L858R and L861Q in exon 21 [32, 34–37]. A background of wild type genomic DNA control in exon 2. In order to detect these mutations using real-time quantitative PCR, the kit utilizes a combination of two different technologies: ARMS and Scorpions. ARMS technology was used for allele-specific amplification, and Scorpions were used to fluorescently signal the PCR products [37, 38]. The Rotor-Gene Q 5plex instrument (Qiagen, Hilden, Germany) was used for the analysis, and the data were collected and analyzed using the Rotor-Gene Q series software following the manufacturer’s instructions.

The Super-ARMS® EGFR Mutation Detection Kit (Amoy Diagnostics, China) was the second kit used in this study. This real-time PCR test is intended to detect 42 somatic mutations in exons 18, 19, 20, and 21 of the EGFR gene in human genomic DNA, including 29 deletions in exon 19, 3 insertions in exon 20, point mutations G719X (exon 18), S768I and T790M (exon 20), and L858R and L861Q (exon 21) [39–41]. The kit employs unique Super-ARMS technology, which has an improved reaction mechanism that can detect even low percentages of mutant EGFR in a wild-type DNA background [40]. It is intended to identify the EGFR mutations, which include 29 deletions in exon 19, 3 insertions in exon 20, point mutations G719X (exon 18), S768I and T790 M (exon 20), and L858R and L861Q (exon 21). The QuantStudio 6 Flex Real-Time PCR machine (Life Technologies, USA) was used to run the PCR program.

Each sample’s mutational status was calculated by measuring the difference in the Ct value between the amplification reactions used for the gene control assay and the mutation-specific assay, as follows: ΔCt = [Ct (mutation-specific assay)–Ct (control assay)] for the therascreen EGFR RGQ PCR Kit, and the mutation status of the sample is recorded as per the cut-off values stated in the manufacturer’s instructions, while in the Super-ARMS EGFR Mutation Detection Kit, the ∆Ct value for each sample is calculated as the following: ∆Ct value = mutant Ct value (FAM/ROX/CY5)–HEX/VIC Ct value, and the mutation status of the sample is called as per the instructions of the manual.

### Statistical analysis

In this study, the data was managed and analyzed using the Statistical Package for the Social Sciences (SPSS) version 26.0. Categorical data was presented as frequencies and percentages of incidence. To compare the frequencies of EGFR mutations in non-small cell lung cancer (NSCLC) patients to those without mutations, the chi-square (*χ*^2^) test was utilized. Furthermore, this test was also used to estimate the relationship between the mutation status and patient characteristics such as gender, age, and smoking status. A significance level of *P* ≤ 0.05 was considered statistically significant.

## Results

### EGFR mutations incidence

**Fig. 1:**
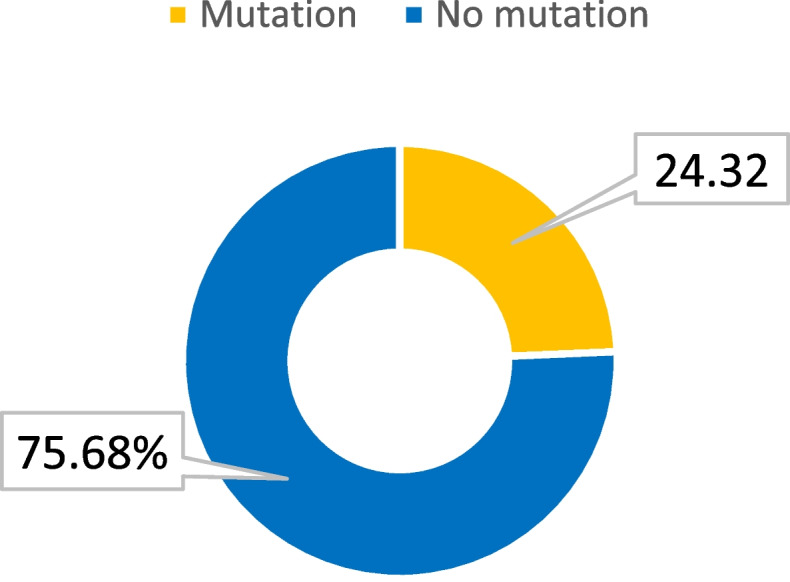
Total incidence of EGFR mutations among all tested subjects

In this study, a group of 333 non-small cell lung cancer (NSCLC) patients were evaluated for clinically relevant EGFR mutations, which included exon 18 point mutations (G719X), exon 19 in-frame deletions, exon 20 point mutations (T790M), and exon 21 point mutations (L858R and L861Q). The findings indicated that 24.32% of the patients (81 out of 333) had EGFR gene mutations, which was statistically significant (*P* < 0.001) when compared to patients without EGFR mutations. Among the EGFR-positive patients, the majority had exon 19 deletion mutations (66.67%), followed by point mutation L858R (28.40%) in exon 21. Only a small proportion of EGFR-positive patients (4.94%) had the TKI-resistant mutation T790M. The remaining 75.68% of patients did not have any identified mutations (Fig. 1) (Table 2).

**Table 2:** Total incidence rate of EGFR mutations among all tested samples and breakdown of the mutations sub-types

**Parameters**		**Mutation**	***N*** **= 333**	**Percentage**	p-value*
Total mutations		No mutation (wild type)	252	75.68%	*P* < 0.001
		Mutations	81	24.32%	
**Parameters**	Exon	Mutation	*N* = 81	Percentage	P-value*
Mutations Break-down	18	G719X	0	0.00%	-
	19	19 deletions	54	66.67%	*P* < 0.001
	20	T790M	4	4.94%	*P* = 0.004
	20	S768I	0	0.00%	-
	20	Insertions	0	0.00%	-
	21	L858R	23	28.40%	*P* < 0.001
	21	L861Q	0	0.00%	-

^*^P-value < 0.05 was considered as significant

### EGFR mutations in association with gender

**Fig. 2:**
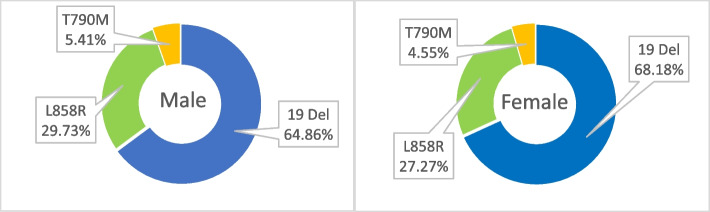
Fractionation EGFR mutations detected according to gender status

EGFR mutations are more common in female than male patients with non-small cell lung cancer. The 81 patients with positive EGFR mutation who were divided into 44 (54.32%) were female, which is a relatively higher proportion than the 37 (45.68%) male patients. This difference in mutation incidence is significant statistically (*P* < 0.001) and shows that gender affects EGFR mutations development in non-small cell lung cancer. The distribution of EGFR mutations was the same in both males and females. The most common mutations were exon 19 deletions and L858R point mutations, and the least common mutation was T790M point mutation. These results suggest that the gender-based difference in EGFR mutation incidence is not due to differences in mutation distribution between males and females. These findings reveal a new insight on how gender affects EGFR mutations development in non-small cell lung cancer patients. The higher prevalence of EGFR mutations in females emphasizes the significance of developing personalized treatment plans for this patient population (Table 3) (Fig. 2).

**Table 3:** EGFR mutations detected according to gender status

	**Male**	**Female**	***p*-value**
Total number (*N* = 333)	230 (69.07%)	103 (30.93%)	*P*-value < 0.001*
Total number of EGFR mutation (*N* = 81)	37 (45.68%)	44 (54.32%)	
19 Del	24 (64.86%)	30 (68.18%)	*P*-value = 0.43
L858R	11 (29.73%)	12 (27.27%)	*P*-value = 0.91
T790M	2 (5.41%)	2 (4.55%)	*P*-value = 0.32

^*^P-value < 0.05 was considered as significant

### EGFR mutations in association with smoking status

**Fig. 3:**
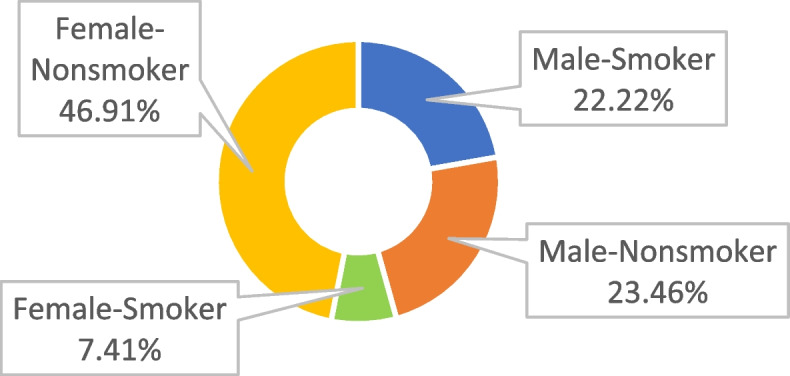
Distribution of the EGFR-positive males and females according to their smoking status

With respect to the smoking status for the study population, almost all the smokers are males, 147 out of the 153 patients (147/153, 96.08%) in comparison to the females 6 out of the 153 patients (6/153, 3.92%). In contrast are the ratios of the nonsmokers where the female patients constitute the larger portion of the nonsmoker numbers 97 patients (97/180, 53.88%), while the number of the nonsmoker male patients is 83 (83/180, 46.12%). With respect to the smoking status relationship with EGFR mutation incidence, it was found that nonsmoking patients were more likely to be with tumors that harbor an activating EGFR mutation (57/81, 70.37%) than smokers (24/81, 29.63%) (57 vs 24, *P* < 0.000 (highly significant)). The highest mutation rate was in the non-smoker females (38 patients out of 57 non-smoker EGFR-positive patients, 66.67%) than the non-smoker males (18 patients out of 57 non-smoker patients, 33.33%) (*P* < 0.000 (highly significant)) (Fig. 3) (Table 4).

**Table 4:** EGFR mutations prevalence in males and females in relation to their respective smoking status

**Smoking status**	**Smoker**	**Nonsmoker**	***p*-value**
**Total number**	153 (45.95%)	180 (54.05%)	
**Number of males**	147 (63.91%)	83 (36.09%)	*P*-value < 0.001*
**Number of female**	6 (5.83%)	97 (94.17%)	
**Total EGFR mutation number**	24 (29.63%)	57 (70.37%)	*P*-value < 0.001*
**EGFR mutations in male**	18 (48.65%)	19 (51.35%)	*P*-value < 0.0001*
**EGFR mutations in female**	6 (13.64%)	38 (86.36%)	

^*^P-value < 0.05 was considered as significant

### EGFR mutations in association of age

Regarding the age factor, although the highest numbers of EGFR-positive mutations patients were observed in the age-groups of 61–80 (40 out of 81 patients, 49.38%) and 41–60 (34 out of 81 patients, 41.98%), there was no statistically significant link found between the gender factor and the age factor. *(P* = 0.096 (no significance)). However, the *P*-value is close to the threshold level of significance (*P* < 0.05), indicating a possible correlation between age ranges and gender. Future studies may need a bigger sample size to prove or disprove this trend. Due to the lack of a statistically significant correlation between EGFR mutation status and age (*P* = 0.934 (no significance)), it appears that there is no significant difference in the prevalence of EGFR mutations across different age groups in this study population. It is possible that this study lacked the power to discern age-specific differences in EGFR mutation incidence. EGFR mutation status does not differ significantly by gender across the different age groups (*P* = 0.504 (no significance)). This indicates that there is no significant gender difference in the prevalence of EGFR mutations in the study population. These findings, while interesting, are based on a small sample size and may not be representative of the population. To validate and broaden these results, and to investigate possible reasons for the observed similarities or differences in EGFR mutation status by gender, more study is needed (Tables 5 and 6).

**Table 5:** Prevalence of EGFR mutations with respect to age and gender

**Age groups**	**20–40**	**41–60**	**61–80**	** > 80**	***p*-value**
Total	23 (6.91%)	140 (42.04%)	163 (48.95%)	7 (2.10%)	
Male	11 (4.78%)	96 (41.74%)	117 (50.87%)	6 (2.61%)	*P*-value = 0.096*
Female	12 (11.65%)	44 (42.72%)	46 (44.66%)	1 (0.97%)	
EGFR mutations total	6 (7.41%)	34 (41.98%)	40 (49.38%)	1 (1.23%)	*P*-value = 0.934*
Male with EGFR mutation	1 (2.70%)	17 (45.95%)	19 (51.35%)	0 (0.00%)	*P*-value = 0.504*
Female with EGFR mutation	5 (11.36%)	17 (38.64%)	21 (47.73%)	1 (2.27%)	

^*^P-value < 0.05 was considered as significant

**Table 6:** A comparison of the demographics of patients from different populations with the current study and the EGFR mutation incidence rate

Study	Ethnicity	Sample size	Age range (median)	Total EGFR mutation (%)	Male/female (%)	Smokers/nonsmokers (%)
**Current study**	**Egyptian**	**333**	**22–89 (61)**	**24.32%**	**69.07%/30.93%**	**45.95%/54.05%**
**Shi et al. (2014) **[14]	**Asian**	**1482**	**17–94 (60)**	**51.4%**	**56.6%/43.4%**	**47.4%/52.6%**
**Tsao et al. (2007) **[42]	**Asian**	**447**	**27–86 (56.5)**	**34.9%**	**55%/45%**	**47.4%/52.6%**
**Hsiao et al. (2014) **[43]	**Asian**	**426**	**NA**	**47%**	**53%/47%**	**57%/43%**
**Sandelin et al. (2015) **[44]	**Western**	**1035**	**NA**	**10%**	**44.6%/55.4%**	**43.3%/56.7%**
**Kauffmann-Guerrero et al. (2018) **[45]	**Western**	**343**	**55–77 (66)**	**17.8%**	**53.9%/46.1%**	**64.4%/35.6%**
**Grosse et al. (2019) **[46]	**Western**	**469**	**53–75 (64)**	**19.2%**	**50.1%/49.9%**	**41.4%/58.6%**
**Gejman et al. (2019) **[47]	**Western**	**300**	**31–85 (65)**	**21.7%**	**36.0%/64%**	**6.9%/93.1%**
**Boustany et al. (2022) **[48]	**Middle East and North Africa**	**6122**	**NA**	**17.2%**	**70.4%/29.6%**	**65.6%/34.4%**
**Benbrahim et al. (2018) **[49]	**Middle East and North Africa**	**1215**	**NA**	**21.2%**	**68.2%/31.8%**	**58.9%/41.1%**

### EGFR mutation incidence across different populations

When comparing the current study finding with other studies that discuss other populations, this comparison developed a table that examines the noteworthy disparities in EGFR mutation rates in non-small cell lung cancer (NSCLC) among ethnicities. While Asian research reveal greater mutation rates (34.9 to 51.4%), our present Egyptian study shows a little lower rate of 24.32%, which is more consistent with Western studies(10–19.2%). These findings highlight the importance of considering regional and demographic factors when evaluating EGFR mutation rates and creating personalizedtreatment plans for NSCLC patients. It emphasizes the need for understanding the specific properties of EGFR mutations in different populations to optimize precision oncology methods.

## Discussion

Lung cancer represents a widespread cancer condition that affects people of all genders [5]. It represents the main cancer-related deaths among males and the second common cause among females, following breast cancer [5, 50]. In Egypt, 5–7% of all malignancies are lung cancers [51]. Although the Middle East has a relatively low risk of developing lung cancer than other parts of the globe with men having a higher prevalence than women [52]. However, the changes in lifestyle and increasing exposure to risk factors, such as smoking and air pollution, are contributing to rise the incidence of lung cancer that are being diagnosed in the Middle East [7]. Lung cancer includes two major categories: non-small cell lung cancer (NSCLC) and small cell lung cancer (SCLC). More than 85% of lung cancer diagnoses are due to NSCLC, whereas the remaining 15% are due to SCLC [20, 53]. There are three subtypes of NSCLC, with adenocarcinoma being the most diagnosed subtype. Large cell carcinoma and squamous cell carcinoma are the other two subtypes [4, 53]. The two primary forms of lung cancer are adenocarcinoma and squamous cell carcinoma, which make up roughly 85% of lung cancer instances [4, 54]. In Egypt, about 40% of lung cancer cases have NSCLC, and about 63% of NSCLC patients have adenocarcinoma [55]. Incidence of lung cancer is largely linked to three factors: gender, age, and smoking status [26]. Men have a higher likelihood of developing lung cancer compared to women, and the risk of developing the disease shows as age dependence [56]. Considering that smoking is the single most significant cause for lung cancer, quitting the habit can significantly lower the likelihood of developing the disease [56]. Exposure to radon, asbestos, and air pollution is additional factors that may increase the lung cancer risk [57]. Early identification and management of lung cancer are crucial for better results and lower mortality rates. Depending on the stage of the disease and the diagnosed lung cancer type, a patient may be recommended to have radiation therapy, chemotherapy, surgery, or a combined set of these approaches for lung cancer [7, 26]. Using biomarkers is essential for lung cancer diagnosis, prognosis, and treatment decisions. Recent improvement within the field of molecular biology has facilitated the detection of a number of driver genes, which are crucial in the initiation and advancement of NSCLC. Among the genes that are considered to be driver genes are the EGFR, KRAS, ALK, and ROS1 [9, 21].

The EGFR mutation is among the well-known biomarkers of the NSCLC that has received the most consideration in recent years [30, 58]. The EGFR mutations incidence is more frequent in Asian populations and adenocarcinoma patients with prevalence up to 50% in Asian populations and up to 10–15% in Caucasian populations [14, 59]. Patients who have EGFR mutations may benefit from targeted therapy, like gefitinib or erlotinib; hence, the existence of EGFR mutations might guide treatment decision-making for these patients [8, 12]. Smokers and squamous cell carcinoma patients are more likely to have KRAS mutations [60, 61], while the EGFR mutations are more frequent in nonsmokers and females [61, 62], and they are linked to an improved response to EGFR-TKIs like erlotinib, gefitinib, and afatinib [8, 56]. KRAS mutations, on the other hand, are present in around 20–30% of cases with NSCLC and have been related with resistance to targeted therapies and chemotherapy, and their presence may be indicative of an inferior prognosis and may limit treatment options [31, 63]. BRAF, in addition to programmed death-ligand 1 (PD-L1), also have been studied in lung cancer [21, 31]. In addition to guiding treatment decisions, biomarkers can be useful for early lung cancer diagnosis and progress screening [60]. Growth, proliferation, and differentiation are all regulated by EGFR, a transmembrane receptor tyrosine kinase [64]. The TK-domain exons 19 and 21 showed the highest mutation rates in relation to NSCLC development, and they are known as EGFR actionable mutations because they predict response to EGFR TKIs [65]. About 90% of all EGFR mutations are attributed to exon 19 partial deletion (19Del) and the L858R mutation in exon 21. These mutations are found most frequently in NSCLC [66, 67]. Exons 18 and 21 have two less frequent mutations [39, 64], and resistance to first- and second-generation TKIs is commonly linked to mutations in exon 20 [68]. Sanger sequencing, real-time PCR, and next-generation sequencing (NGS) are only few of the technologies that can be employed to identify EGFR mutations in NSCLC [24, 31]. Real-time PCR is an extremely precise and sensitive method that can identify EGFR mutations in as low as 1% of tumor cells [69, 70].

In the current study, the study population consists of 333 tumor samples subjected to screening for EGFR gene mutations using specific probes. The relation among the status of the EGFR mutation and three elemental factors was studied: EGFR mutation status vs. gender, age, and smoking status, which are considered direct contributing factors to lung cancer incidence [71]. In our study, 333 subjects were diagnosed with NSCLC and were included. When testing the paraffin blocks of the included samples, 81 (24.32%) of the 333 patients (*P* < 0.001) were found to harbor EGFR-positive mutation. The incidence is lower than that reported in the data published from the PIONEER trial, which revealed a greater prevalence among Asians (51.4%). Also, the incidence of the EGFR mutations was found to be greater in women, never smokers, and patients with an earlier disease stage [14]. In PIONEER trial, EGFR mutations were found to have a correlation with factors such as disease stage, country, ethnicity origin, gender, histological type, smoking status, and pack-years, additionally indicating that the EGFR mutations incidence rate was greater in certain Asian countries, with Thailand having the highest prevalence (46.2%), and the lowest was in India (11.5%) [14]. It was also reported in another study that in Asian patients, EGFR mutations were found to be more prevalent (48%) which is substantially greater than Caucasian patients (10%) [72] and higher than the incidence rate of the current study. Another meta-analysis of patient data from six medical centers in Mainland China found that the total ratio of EGFR mutations in that cohort was 30.04%, which is comparable to the EGFR mutation ratio observed in this study [73]. In a study with a Polish population, the percentage of EGFR mutation-positive subjects was 20.5%, which is quite similar to our study’s prevalence of EGFR mutation [74]. According to Lindeman et al. (2018)’s recommendation, among Western NSCLC patients, EGFR mutations are present in 10–15% [31]. The International Association for the Study of Lung Cancer (IASLC) declared the occurrence of EGFR mutations in 10–20% of NSCLC patients in Western populations [75]; both studies report a lower EGFR mutations frequency than the rate reported by current study. It was observed that the frequency of EGFR mutations was higher in patients from the Middle East and Africa than it was in patients from white populations, but it was lower than it was in Asian ethnicities where the overall rate of EGFR mutations was 21.2% in a review study across 10 studies including Middle Eastern countries as Egypt, Turkey, Lebanon, Saudi Arabia, UAE, and Morocco [49]. The EGFR mutation incidence nearly agrees with the results from the current study. There were two other review studies that reported the EGFR mutation-positive incidence in Middle Eastern and African populations. The first one reported that the mutation rate in the region was 36.9% [58], which is higher than our study’s rate of 24.32%; however, the difference could be attributed to the included sample size of 17,026 newly diagnosed patients in different countries in the gulf region and the method of EGFR mutation detection employed in that study. The second study involved a systematic reviewing for the literature to assess the prevalence of EGFR mutations and their relation to ethnicity and clinicopathological factors in patients with NSCLC from the North Africa and Middle East. The review comprised 24 studies that examined EGFR mutations in 6544 NSCLC patients [48]. The EGFR mutations with the highest overall prevalence were deletions of exon 19 and L858R substitution in exon 21, both of which had a percentage of 17.9%. According to the review, EGFR mutations were increasingly common among nonsmokers, females, and with adenocarcinoma diagnosis. The NSCLC prevalence in MENA populations is slightly greater than in Caucasian NSCLC patients, but it is lower than in Asian NSCLC patients [48], a finding slightly lower than the reported EGFR mutation-positive rate in current study. In Egypt, a peak study investigation of a population of 2017 study participants revealed that the incidence of EGFR mutation was 17.5%, which is comparable to the ranges reported in Middle-East studies but lower than the incidence rate reported in the present study (24.32%) [76]. Two other Egyptian studies investigated the relationship between the EGFR incidence and other biomarkers such as KRAS and MET as other factors contributing to the development of NSCLC [77]. In the other study, the incidence relationship between EGFR and MET mutations was examined, and it was found that 25% of NSCLC patients had EGFR mutations and 10% had MET mutations [78]; both studies reported an EGFR incidence agrees with the current study.

Previous studies discovered a correlation between EGFR-positive mutations and adenocarcinoma histology, never-smoking status, and female gender [56, 79, 80]. Females exhibited a greater frequency of EGFR mutations than males in the current study population (54.32% vs. 45.68%,* P* < 0.001, highly significant). This conclusion was consistent with the incidence rates observed in Asians, where females were found to have an increased rate of EGFR mutations compared to males (61.1% [384/628] of the total population) than males (44.0% [362/822]) [14]. Also in the Middle East and Africa, EFGR mutations are present in females more than in males [49, 81, 82]. In studies that included Egyptian participants, it was found that the EGFR mutation incidence is greater in females than in males [48, 51, 76, 77, 83, 84]. According to the findings of another study, the prevalence of EGFR mutations in females is significantly higher than it is in males (71.4% vs. 28.6%, respectively) [85]. In the current study, the link between EGFR mutation incidence and smoking status revealed that nonsmokers had higher EGFR-positive mutations than those who smoked (70.37% vs. 29.63%, respectively, *P* < 0.001, highly significant). Smoking was found to be correlated with a lower incidence of mutation rate in EGFR gene in patients with NSCLC in studies involving many ethnic groups [49, 71, 81, 85], and that poor prognosis in smoking patients diagnosed with NSCLC is related to lower rates of mutations in EGFR gene [60, 86]. In Egypt, two different studies reported an incidence rate of EGFR mutations among smokers and nonsmokers that was comparable to the findings of the current study [83, 85].

In developed countries, the average age of individuals diagnosed with lung cancer is 60 years old while in the study’s population has averaged around 50 years old. At lower ages (7.41%) and older ages above 80 (1.23%), the development of EGFR mutations is rather small. Most of the study population was between the ages of 40–60 (41.98%) and 61–80 (49.38%). No significant correlation has existed between the age factor and gender (*P* = 0.096 (no significance)). On the other hand, the *P*-value is quite close to the threshold level of significance (*P* < 0.05), which indicates that there may be a correlation between age ranges and gender. In further studies, it will likely be necessary to use a bigger sample size to validate or invalidate this association. As a result of the absence of a correlation that is significant statistically between the existence of EGFR mutations and age (*P* = 0.934 (no significance)), there is no significant differences in the prevalence of EGFR mutations across various age groups in the population that was used for this study. It is probable that this study did not have enough power to distinguish between age-specific changes regarding the incidence of EGFR mutations. The occurrence or absence of an EGFR mutation does not differ significantly between males and females across the various age groups (*P* = 0.504 (no significance)). This suggests that there is no discernible difference between the existence of EGFR mutations in men and women among the participants in the research population. While these findings are based on a small sample size, it is possible that they are not indicative of the population. More research is required to evaluate and expand upon these findings, as well as to understand the underlying causes of any observed similarities or variations in the EGFR mutation status according to gender. Despite the fact that a number of studies have discovered that the age factor and the prevalence of EGFR mutations are significantly correlated, other studies have found no such association. It is essential to note, however, that the sample sizes in each age group may have influenced the results, and that in order to further examine the correlation between age and the incidence of EGFR mutations, it is possible that larger studies are required. According to one study, older age was associated with an increased incidence rate of EGFR mutations in lung cancer patients. The study revealed that patients over the median age of 57 were more likely than younger patients to have EGFR mutations [87]. However, the results of two studies exhibited the absence of the significant correlation between lung cancer patients’ ages and the frequency of EGFR mutations [88, 89].

According to the IPASS (IRESSA Pan-Asian Study) research [90], approximately 96% harbored TKI-sensitive mutations in EGFR gene in either exon 21 or exon 19 [90]. Similarly, the incidence of such mutations was reported to be 95.07% in the current study, 66.67% for exon 19 deletions (*P* < 0.001 (highly significant)) and 28.40% (*P* < 0.001 (highly significant)) for the L858R point mutation. According to the findings of a different investigation, the two mutations that were found to be the most common were deletions in exon 19 (45%) and L858R in exon 21 (40%), which differs marginally from the incidence rates reported in the current study for 19 deletions and L858R [79]. In contrast to this, Zaki et al. [70] reported exon 19 deletions in 22% of NSCLC patients in an earlier Egyptian study of 50 cases. This percentage is comparable to the incidence among Caucasians [77] or East Asians [78]. Nevertheless, none of the cases carried the L858R point mutation in exon 21 [70]. The L858R point mutation occurs in 16–46% of East Asian patients with NSCLC, indicating that the East Asian population has a higher incidence of this mutation than other populations [91]. The T790M mutation is a point mutation that occurs at codon 790 of exon 20 of the EGFR gene that replaces threonine with methionine [92]. This mutation accounts for 4.94% (4/81, *P* = 0.004 (significant)) of our mutated cases, which is consistent with previous conclusions indicating that depending on the demographic and the sample size, the T790M mutation occurs in 1–17% of all EGFR mutations [93, 94]. Its presence is associated with progression of the disease and a poor outcome [94–99]. According to the findings of one study, the T790M mutation is the most important factor in TKI resistance [94, 100]. The majority of the research that investigated the recognition of the T790M mutation focused on patients who had shown progression after receiving treatment with an EGFR TKI [93, 94, 101, 102]. Because the majority of the T790M mutation patients were resistant to gefitinib, erlotinib, or afatinib without suppressing the growth of the tumor in NSCLC [93, 103, 104], TKIs of the third generation, such as osimertinib (Tagrisso), which are designed to overcome attained resistance to TKIs, have demonstrated encouraging therapeutic outcomes [66, 105] Gefitinib was more efficient than chemotherapy in treating EGFR mutations in patients, particularly those with point mutation L858R in exon 21 and deletions in exon 19. Patients who had exon 19 deletions had a significantly greater in response rate than those with L858R mutations [99, 106]. With respect to smoking status, the correlation between the incidence of mutations in EGFR gene and smoking status has been the subject of debate in the scientific community. EGFR mutations incidence have been linked to being a nonsmoker in a number of studies. In the current study, nonsmokers had EGFR mutations at a higher rate 70.37% than smokers (29.63%) (*P* < 0.000); also, it was found that the non-smoker females had a higher mutation rate 66.67% than the non-smoker males 33.33% (*P* < 0.000 (very significant)). These findings are consistent with prior studies that mentioned the same association between smoking and EGFR mutations incidence [25, 56, 73, 86, 107]. In a meta-analysis including 26 studies and 3688 NSCLC patients, Ren et al. revealed that nonsmokers had a considerably increased rate of EGFR mutations [108]. Several studies show that nonsmokers, notably females, are more likely than smokers to have tumors with activating EGFR mutations [56, 83, 85, 109]. The mechanism underlying the correlation between nonsmoking status and EGFR mutations is incompletely understood. It has been hypothesized, however, that exposure to tobacco smoke may result in the accumulation of other genetic alterations that can obscure the effect of EGFR mutations [34, 110]. The association between nonsmoking and the incidence of EGFR mutations has significant implications for treating lung cancer patients [8, 13, 111]. EGFR-TKIs as gefitinib and erlotinib have a higher response rate in patients with EGFR mutations compared to patients without mutations [8, 73, 112]. The effectiveness of EGFR TKIs in treating lung cancer patients with EGFR mutations emphasizes the significance of molecular profiling in selecting targeted therapies for those patients, and so identifying EGFR mutations patients is crucial for determining the optimal treatment. The development of precision oncology has irreversibly changed the landscape of cancer treatment. Unlike previous “one-size-fits-all” approaches, this new system tailors therapies to individual patients based on their unique genetic profile [28]. The identification of epidermal growth factor receptor (EGFR) mutations caused a paradigm shift in the field of non-small cell lung cancer (NSCLC) [28]. Recognizing the importance of these mutations in therapy success, regular EGFR testing has become standard practice in NSCLC care [27]. Routine EGFR testing is critical in non-small cell lung cancer (NSCLC) since it is a predictive biomarker for targeted therapy [113]. It is critical to investigate future paths in precision oncology testing of EGFR in NSCLC, including current challenges, developments in testing technologies, personalized treatment methods, overcoming resistance to EGFR targeted therapies, and establishing routine EGFR testing [30, 113]. In the present landscape, available methodologies and techniques for EGFR testing in NSCLC include tissue-based testing using techniques such as PCR and sequencing. However, these approaches have drawbacks, including sample availability, tumor heterogeneity, and turnaround time [30, 48]. Tissue biopsies are still considered the gold standard, although their invasive nature limits their use [31]. Tumor heterogeneity poses a challenge for accurate EGFR testing due to probable missing mutations, which can result in underrepresentation of significant mutations found in different tumor regions or the failure to detect uncommon mutations and acquired resistance mechanisms [101, 114]. Accurate EGFR testing is critical for making personalized treatment decisions by detecting specific mutations and directing targeted therapies. Furthermore, the discovery of uncommon EGFR mutations and the possibility of combining EGFR testing with additional indicators improve therapy effectiveness [30]. Advancements in EGFR testing technologies have significantly impacted precision oncology, especially in NSCLC, aimed to address the limitations of traditional testing methods by offering more efficient and comprehensive methods for detecting EGFR alterations in tumors [115]. One major innovation is the introduction of liquid biopsies as a noninvasive and effective method of EGFR testing. Liquid biopsies involve the study of circulating tumor DNA (ctDNA) in the blood, which allows for the discovery of genetic variations such as EGFR mutations without the need for invasive tissue biopsy. This method is especially useful in cases when obtaining tissue samples is difficult, since it allows for a more dynamic assessment of tumor genetic alterations over time, providing insights into tumor progression and treatment response [75]. Next-generation sequencing (NGS) technologies have also transformed EGFR testing by enabling thorough genomic profiling of EGFR mutations [116]. NGS enables the simultaneous investigation of many genes and genetic mutations, resulting in a more detailed understanding of the tumor’s genomic landscape [117, 118]. In the context of EGFR testing in NSCLC, NGS can reveal both common and unusual EGFR mutations, which may have consequences for therapy decisions. Given the heterogeneity found in NSCLC tumors, this thorough technique is critical for detecting the complete range of EGFR mutations [117, 118]. Digital pathology and artificial intelligence (AI) have significantly improved the accuracy and speed of EGFR tests [119]. Digital pathology is the digitalization of histopathology slides, which enables remote access and study of tissue samples [119]. AI-based algorithms can help pathologists comprehend complicated genomic data, identify particular genetic mutations, and assess tumor heterogeneity more efficiently [119, 120]. Using AI, healthcare practitioners may improve the precision and consistency of EGFR testing findings, resulting in better informed treatment decisions for NSCLC patients [119, 120]. Resistance to EGFR-targeted treatments continues to be a substantial concern in NSCLC [112]. In conclusion, the routine EGFR testing is critical for tracking treatment outcomes and discovering resistance mechanisms early on [121]. This enables clinicians to modify treatment plans before disease development, potentially prolonging patient longevity and enhancing quality of life [101]. Future research will focus on creating innovative treatment techniques, such as second generation and beyond TKIs with wider inhibitory efficacy and combination medicines that target alternative resistance pathways [122, 123].

## Conclusion

Across all demographics, EGFR mutations are still regarded as the most prevalent actionable alterations in NSCLC. Despite the fact that the frequency of EGFR mutations varies by population, these mutations remain the most prevalent actionable mutations in NSCLC patients. The primary objective of the present study was to identify the prevalence of frequently occurring actionable EGFR gene mutations and examine their association with gender, smoking status, and age in the Egyptian population. It was discovered that the incidence frequency of EGFR mutations is highly correlated with smoking status and gender, but not with age. According to the results of this investigation, EGFR mutation testing should be performed on NSCLC patients in Egypt due to the high prevalence of tumor EGFR mutation identified. Patients with EGFR-mutated tumor may benefit from this technique, which should improve the accurate diagnosis and treatment. Previous studies have documented an increased incidence of EGFR mutations within female and non-smoker NSCLC patients, which is supported by our findings. To guide personalized treatment decisions, EGFR mutation screening should be considered for NSCLC patients, particularly females and nonsmokers, based on these findings. Our study has a number of limitations, including a retrospective design and a paucity of information regarding treatment outcomes. Nevertheless, our findings emphasize the significance of EGFR mutation testing in NSCLC patients, particularly in females and nonsmokers. Early detection of EGFR mutations can guide personalized treatment decisions and enhance NSCLC patient survival. Additional research is required to validate our findings and investigate the impact of testing for EGFR mutations on the outcomes of treatment in Egyptian NSCLC patients.

## Supplementary Information

Additional file 1: Table S1. The Age, Smoking status and Gender for the study subjects and their corresponding detected EGFR mutations.

## Data Availability

All data generated or analyzed during this study are included in this published article and its supplementary information file (Table S1).

## Abbreviations

EGFR: Epidermal growth factor receptor oncogene
ESMO: European Society for Medical Oncology
FFPE: Formalin-fixed paraffin-embedded
GCP: Good clinical practice
IASLC: International Association for the Study of Lung Cancer
IPASS: Iressa Pan-Asia Study
ROS1: C-ros oncogene 1
ALK: Anaplastic lymphoma kinase oncogene
KRAS: Kristen rat sarcoma viral oncogene
MENA: Middle East/North Africa
MET: Mesenchymal-epithelial transition oncogene
NCCN: National Comprehensive Cancer Network
NGS: Next-generation sequencing
NSCLC: Non-small cell lung cancer
SCLC: Small cell lung cancer
TKI: Tyrosine kinase inhibitor
WHO: World Health Organization
ARMS: Amplified refractory mutation system
LUAD: Lung adenocarcinoma
ASR: Age-specific incidence rates
