# Supplementary material for: The prevalence and clinical significance of EGFR mutations in non-small cell lung cancer patients in Egypt: a screening study
Source: J Egypt Natl Canc Inst. 2024 Dec 23;36:39. doi: 10.1186/s43046-024-00251-1 (PMC13313845; doi:10.1186/s43046-024-00251-1)
Supplement: Supplementary file 1 — Additional file 1: Table S1. The Age, Smoking status and Gender for the study subjects and their corresponding detected EGFR mutations. [file 43046_2024_251_MOESM1_ESM.docx]

**Title: The Prevalence and Clinical Significance of EGFR Mutations in Non-Small Cell Lung Cancer Patients in Egypt: A Screening Study**

**Table S1:** The Age, Smoking status and Gender for the study subjects and their corresponding detected EGFR mutations

| Subject ID | Age | Smoking | Results | Gender |
| --- | --- | --- | --- | --- |
| 1 | 49 | *yes* | No mutation detected | male |
| 2 | 50 | *yes* | 19 Deletions in exon 19 | female |
| 3 | 67 | *yes* | 19 Deletions in exon 19 | female |
| 4 | 65 | *yes* | No mutation detected | male |
| 5 | 70 | *yes* | No mutation detected | male |
| 6 | 71 | *yes* | No mutation detected | male |
| 7 | 62 | *no* | 19 Deletions in exon 19 | female |
| 8 | 60 | *yes* | No mutation detected | male |
| 9 | 52 | *no* | 19 Deletions in exon 19 | female |
| 10 | 66 | *no* | No mutation detected | female |
| 11 | 50 | *no* | No mutation detected | female |
| 12 | 66 | *yes* | No mutation detected | male |
| 13 | 70 | *no* | No mutation detected | male |
| 14 | 48 | *no* | No mutation detected | male |
| 15 | 62 | *no* | No mutation detected | female |
| 16 | 74 | *yes* | No mutation detected | male |
| 17 | 62 | *yes* | T790M in exon 20 | male |
| 18 | 59 | *no* | No mutation detected | male |
| 19 | 64 | *yes* | No mutation detected | male |
| 20 | 47 | *yes* | No mutation detected | male |
| 21 | 68 | *no* | No mutation detected | male |
| 22 | 65 | *yes* | No mutation detected | male |
| 23 | 44 | *yes* | No mutation detected | male |
| 24 | 58 | *no* | No mutation detected | male |
| 25 | 54 | *yes* | No mutation detected | male |
| 26 | 46 | *no* | No mutation detected | female |
| 27 | 83 | *no* | No mutation detected | male |
| 28 | 74 | *no* | No mutation detected | male |
| 29 | 71 | *no* | No mutation detected | female |
| 30 | 58 | *no* | No mutation detected | male |
| 31 | 57 | *no* | No mutation detected | male |
| 32 | 67 | *yes* | No mutation detected | male |
| 33 | 65 | *yes* | No mutation detected | male |
| 34 | 45 | *yes* | No mutation detected | male |
| 35 | 57 | *no* | No mutation detected | female |
| 36 | 57 | *yes* | No mutation detected | male |
| 37 | 63 | *no* | No mutation detected | female |
| 38 | 63 | *yes* | No mutation detected | male |
| 39 | 70 | *no* | No mutation detected | male |
| 40 | 63 | *yes* | No mutation detected | male |
| 41 | 62 | *yes* | No mutation detected | male |
| 42 | 60 | *no* | 19 Deletions in exon 19 | female |
| 43 | 78 | *no* | No mutation detected | male |
| 44 | 54 | *yes* | No mutation detected | male |
| 45 | 70 | *no* | No mutation detected | male |
| 46 | 34 | *yes* | No mutation detected | male |
| 47 | 47 | *no* | 19 Deletions in exon 19 | male |
| 48 | 61 | *yes* | No mutation detected | male |
| 49 | 58 | *no* | No mutation detected | male |
| 50 | 71 | *no* | 19 Deletions in exon 19 | female |
| 51 | 49 | *yes* | No mutation detected | male |
| 52 | 72 | *yes* | No mutation detected | male |
| 53 | 55 | *yes* | No mutation detected | male |
| 54 | 63 | *yes* | No mutation detected | male |
| 55 | 41 | *yes* | No mutation detected | male |
| 56 | 62 | *no* | No mutation detected | male |
| 57 | 68 | *no* | No mutation detected | female |
| 58 | 43 | *no* | No mutation detected | male |
| 59 | 62 | *no* | No mutation detected | male |
| 60 | 55 | *yes* | 19 Deletions in exon 19 | Female |
| 61 | 40 | *yes* | 19 Deletions in exon 19 | Female |
| 62 | 63 | *yes* | No mutation detected | male |
| 63 | 67 | *no* | 19 Deletions in exon 19 | female |
| 64 | 50 | *no* | No mutation detected | male |
| 65 | 64 | *no* | No mutation detected | male |
| 66 | 70 | *no* | No mutation detected | male |
| 67 | 72 | *yes* | No mutation detected | male |
| 68 | 49 | *yes* | No mutation detected | male |
| 69 | 69 | *no* | No mutation detected | female |
| 70 | 66 | *no* | L858R IN EXON 21 | female |
| 71 | 70 | *no* | 19 Deletions in exon 19 | male |
| 72 | 65 | *yes* | No mutation detected | male |
| 73 | 50 | *no* | No mutation detected | male |
| 74 | 74 | *yes* | No mutation detected | male |
| 75 | 59 | *yes* | No mutation detected | male |
| 76 | 63 | *yes* | No mutation detected | male |
| 77 | 60 | *no* | L858R IN EXON 21 | Female |
| 78 | 59 | *no* | No mutation detected | female |
| 79 | 77 | *no* | No mutation detected | female |
| 80 | 64 | *no* | No mutation detected | female |
| 81 | 77 | *no* | 19 Deletions in exon 19 | male |
| 82 | 40 | *yes* | No mutation detected | male |
| 83 | 65 | *no* | No mutation detected | female |
| 84 | 57 | *no* | No mutation detected | female |
| 85 | 64 | *yes* | No mutation detected | male |
| 86 | 65 | *yes* | 19 Deletions in exon 19 | female |
| 87 | 47 | *yes* | No mutation detected | male |
| 88 | 37 | *no* | No mutation detected | female |
| 89 | 54 | *yes* | No mutation detected | male |
| 90 | 31 | *no* | No mutation detected | female |
| 91 | 78 | *yes* | No mutation detected | male |
| 92 | 35 | *no* | No mutation detected | male |
| 93 | 68 | *yes* | No mutation detected | male |
| 94 | 70 | *yes* | No mutation detected | male |
| 95 | 66 | *yes* | No mutation detected | male |
| 96 | 72 | *no* | No mutation detected | male |
| 97 | 76 | *no* | L858R IN EXON 21 | female |
| 98 | 46 | *no* | No mutation detected | male |
| 99 | 49 | *no* | No mutation detected | female |
| 100 | 49 | *no* | No mutation detected | male |
| 101 | 67 | *no* | No mutation detected | female |
| 102 | 69 | *yes* | No mutation detected | male |
| 103 | 61 | *no* | No mutation detected | male |
| 104 | 54 | *no* | 19 Deletions in exon 19 | female |
| 105 | 63 | *no* | 19 Deletions in exon 19 | female |
| 106 | 67 | *no* | No mutation detected | female |
| 107 | 32 | *no* | No mutation detected | female |
| 108 | 67 | *yes* | No mutation detected | male |
| 109 | 62 | *yes* | No mutation detected | male |
| 110 | 71 | *no* | No mutation detected | male |
| 111 | 70 | *no* | L858R IN EXON 21 | male |
| 112 | 47 | *yes* | No mutation detected | male |
| 113 | 75 | *no* | No mutation detected | male |
| 114 | 74 | *no* | L858R IN EXON 21 | female |
| 115 | 51 | *yes* | No mutation detected | male |
| 116 | 55 | *no* | No mutation detected | male |
| 117 | 65 | *yes* | No mutation detected | male |
| 118 | 63 | *no* | No mutation detected | male |
| 119 | 50 | *no* | No mutation detected | female |
| 120 | 48 | *yes* | No mutation detected | male |
| 121 | 56 | *no* | No mutation detected | female |
| 122 | 63 | *yes* | No mutation detected | male |
| 123 | 73 | *no* | No mutation detected | female |
| 124 | 52 | *yes* | No mutation detected | male |
| 125 | 57 | *yes* | No mutation detected | male |
| 126 | 70 | *no* | No mutation detected | male |
| 127 | 58 | *no* | No mutation detected | male |
| 128 | 64 | *no* | No mutation detected | male |
| 129 | 47 | *no* | No mutation detected | male |
| 130 | 32 | *no* | No mutation detected | female |
| 131 | 69 | *no* | No mutation detected | male |
| 132 | 40 | *no* | L858R IN EXON 21 | female |
| 133 | 50 | *yes* | No mutation detected | male |
| 134 | 60 | *yes* | No mutation detected | male |
| 135 | 59 | *yes* | No mutation detected | male |
| 136 | 65 | *no* | No mutation detected | female |
| 137 | 71 | *no* | No mutation detected | female |
| 138 | 61 | *yes* | No mutation detected | male |
| 139 | 62 | *yes* | No mutation detected | male |
| 140 | 69 | *yes* | No mutation detected | male |
| 141 | 49 | *yes* | No mutation detected | male |
| 142 | 56 | *no* | 19 Deletions in exon 19 | female |
| 143 | 65 | *no* | No mutation detected | male |
| 144 | 68 | *no* | No mutation detected | male |
| 145 | 42 | *yes* | No mutation detected | male |
| 146 | 70 | *no* | No mutation detected | male |
| 147 | 54 | *yes* | 19 Deletions in exon 19 | male |
| 148 | 71 | *no* | 19 Deletions in exon 19 | female |
| 149 | 45 | *no* | No mutation detected | female |
| 150 | 82 | *yes* | No mutation detected | male |
| 151 | 68 | *yes* | No mutation detected | male |
| 152 | 50 | *no* | No mutation detected | female |
| 153 | 57 | *no* | No mutation detected | female |
| 154 | 62 | *yes* | L858R IN EXON 21 | male |
| 155 | 72 | *yes* | No mutation detected | male |
| 156 | 40 | *no* | No mutation detected | female |
| 157 | 40 | *no* | No mutation detected | male |
| 158 | 44 | *no* | no mutation detected | female |
| 159 | 31 | *yes* | No mutation detected | male |
| 160 | 63 | *no* | No mutation detected | female |
| 161 | 64 | *yes* | No mutation detected | male |
| 162 | 46 | *yes* | L858R IN EXON 21 | male |
| 163 | 59 | *no* | No mutation detected | female |
| 164 | 49 | *no* | 19 Deletions in exon 19 | female |
| 165 | 61 | *yes* | No mutation detected | male |
| 166 | 43 | *no* | T790M in exon 20 | female |
| 167 | 54 | *no* | 19 Deletions in exon 19 | female |
| 168 | 61 | *no* | 19 Deletions in exon 19 | female |
| 169 | 52 | *no* | 19 Deletions in exon 19 | female |
| 170 | 73 | *yes* | No mutation detected | male |
| 171 | 51 | *yes* | No mutation detected | male |
| 172 | 46 | *no* | No mutation detected | female |
| 173 | 58 | *no* | 19 Deletions in exon 19 | male |
| 174 | 22 | *no* | No mutation detected | male |
| 175 | 50 | *yes* | No mutation detected | male |
| 176 | 63 | *no* | 19 Deletions in exon 19 | female |
| 177 | 35 | *no* | No mutation detected | male |
| 178 | 63 | *no* | No mutation detected | male |
| 179 | 49 | *yes* | No mutation detected | male |
| 180 | 49 | *no* | L858R IN EXON 21 | male |
| 181 | 66 | *yes* | No mutation detected | male |
| 182 | 66 | *no* | No mutation detected | male |
| 183 | 49 | *yes* | No mutation detected | male |
| 184 | 52 | *no* | No mutation detected | female |
| 185 | 67 | *no* | L858R IN EXON 21 | female |
| 186 | 55 | *no* | L858R IN EXON 21 | male |
| 187 | 54 | *no* | No mutation detected | female |
| 188 | 65 | *no* | No mutation detected | female |
| 189 | 56 | *yes* | No mutation detected | male |
| 190 | 70 | *yes* | No mutation detected | male |
| 191 | 47 | *yes* | No mutation detected | male |
| 192 | 60 | *yes* | No mutation detected | male |
| 193 | 69 | *no* | No mutation detected | male |
| 194 | 58 | *no* | T790M in exon 20 | male |
| 195 | 68 | *yes* | No mutation detected | male |
| 196 | 60 | *yes* | No mutation detected | male |
| 197 | 54 | *no* | No mutation detected | male |
| 198 | 61 | *yes* | No mutation detected | male |
| 199 | 56 | *no* | L858R IN EXON 21 | female |
| 200 | 40 | *yes* | No mutation detected | male |
| 201 | 52 | *yes* | No mutation detected | male |
| 202 | 53 | *yes* | No mutation detected | male |
| 203 | 53 | *no* | no mutation detected | male |
| 204 | 63 | *no* | L858R IN EXON 21 | male |
| 205 | 54 | *no* | No mutation detected | female |
| 206 | 52 | *no* | No mutation detected | male |
| 207 | 70 | *yes* | 19 Deletions in exon 19 | male |
| 208 | 86 | *no* | L858R IN EXON 21 | female |
| 209 | 70 | *yes* | No mutation detected | male |
| 210 | 61 | *yes* | L858R IN EXON 21 | male |
| 211 | 55 | *yes* | L858R IN EXON 21 | male |
| 212 | 44 | *yes* | No mutation detected | male |
| 213 | 65 | *yes* | No mutation detected | male |
| 214 | 25 | *no* | 19 Deletions in exon 19 | female |
| 215 | 73 | *no* | 19 Deletions in exon 19 | female |
| 216 | 74 | *yes* | No mutation detected | male |
| 217 | 63 | *yes* | no mutation detected | male |
| 218 | 70 | *yes* | No mutation detected | male |
| 219 | 72 | *no* | No mutation detected | male |
| 220 | 63 | *no* | L858R IN EXON 21 | female |
| 221 | 58 | *yes* | No mutation detected | male |
| 222 | 68 | *no* | No mutation detected | female |
| 223 | 59 | *yes* | No mutation detected | male |
| 224 | 71 | *yes* | No mutation detected | male |
| 225 | 66 | *yes* | No mutation detected | male |
| 226 | 63 | *no* | No mutation detected | female |
| 227 | 64 | *yes* | No mutation detected | male |
| 228 | 52 | *no* | No mutation detected | female |
| 229 | 44 | *yes* | No mutation detected | male |
| 230 | 30 | *no* | No mutation detected | female |
| 231 | 65 | *yes* | No mutation detected | male |
| 232 | 56 | *no* | No mutation detected | female |
| 233 | 34 | *no* | No mutation detected | female |
| 234 | 89 | *yes* | No mutation detected | male |
| 235 | 45 | *no* | No mutation detected | female |
| 236 | 62 | *no* | No mutation detected | female |
| 237 | 74 | *no* | No mutation detected | male |
| 238 | 40 | *no* | 19 Deletions in exon 19 | female |
| 239 | 58 | *no* | 19 Deletions in exon 19 | female |
| 240 | 65 | *yes* | No mutation detected | male |
| 241 | 60 | *no* | 19 Deletions in exon 19 | male |
| 242 | 58 | *no* | No mutation detected | male |
| 243 | 70 | *no* | No mutation detected | female |
| 244 | 74 | *no* | 19 Deletions in exon 19 | male |
| 245 | 54 | *no* | no mutation detected | female |
| 246 | 55 | *yes* | No mutation detected | male |
| 247 | 50 | *yes* | 19 Deletions in exon 19 | male |
| 248 | 59 | *no* | No mutation detected | female |
| 249 | 72 | *no* | No mutation detected | female |
| 250 | 60 | *yes* | No mutation detected | male |
| 251 | 53 | *yes* | 19 Deletions in exon 19 | male |
| 252 | 60 | *yes* | No mutation detected | male |
| 253 | 72 | *yes* | No mutation detected | male |
| 254 | 83 | *yes* | No mutation detected | male |
| 255 | 67 | *no* | L858R IN EXON 21 | female |
| 256 | 68 | *yes* | 19 Deletions in exon 19 | male |
| 257 | 47 | *yes* | 19 Deletions in exon 19 | female |
| 258 | 51 | *yes* | No mutation detected | male |
| 259 | 78 | *no* | L858R IN EXON 21 | female |
| 260 | 56 | *no* | No mutation detected | male |
| 261 | 73 | *no* | No mutation detected | female |
| 262 | 63 | *no* | No mutation detected | male |
| 263 | 58 | *no* | No mutation detected | male |
| 264 | 72 | *yes* | No mutation detected | male |
| 265 | 45 | *no* | No mutation detected | male |
| 266 | 60 | *no* | 19 Deletions in exon 19 | female |
| 267 | 81 | *yes* | No mutation detected | male |
| 268 | 65 | *no* | L858R IN EXON 21 | male |
| 269 | 63 | *no* | No mutation detected | male |
| 270 | 43 | *no* | No mutation detected | female |
| 271 | 62 | *no* | No mutation detected | female |
| 272 | 62 | *yes* | No mutation detected | male |
| 273 | 72 | *yes* | No mutation detected | male |
| 274 | 46 | *no* | No mutation detected | male |
| 275 | 69 | *no* | L858R IN EXON 21 | Female |
| 276 | 53 | *yes* | No mutation detected | male |
| 277 | 73 | *yes* | No mutation detected | male |
| 278 | 53 | *yes* | No mutation detected | male |
| 279 | 49 | *no* | 19 Deletions in exon 19 | male |
| 280 | 58 | *yes* | No mutation detected | male |
| 281 | 67 | *no* | 19 Deletions in exon 19 | male |
| 282 | 74 | *yes* | 19 Deletions in exon 19 | male |
| 283 | 58 | *yes* | No mutation detected | male |
| 284 | 60 | *yes* | 19 Deletions in exon 19 | male |
| 285 | 59 | *yes* | 19 Deletions in exon 19 | male |
| 286 | 79 | *yes* | No mutation detected | male |
| 287 | 65 | *yes* | No mutation detected | male |
| 288 | 64 | *no* | no mutation detected | male |
| 289 | 70 | *no* | 19 Deletions in exon 19 | female |
| 290 | 65 | *no* | No mutation detected | male |
| 291 | 62 | *no* | No mutation detected | male |
| 292 | 62 | *yes* | No mutation detected | male |
| 293 | 72 | *yes* | 19 Deletions in exon 19 | male |
| 294 | 57 | *no* | No mutation detected | male |
| 295 | 51 | *no* | No mutation detected | male |
| 296 | 60 | *no* | No mutation detected | female |
| 297 | 60 | *no* | No mutation detected | male |
| 298 | 66 | *yes* | 19 Deletions in exon 19 | male |
| 299 | 60 | *yes* | 19 Deletions in exon 19 | male |
| 300 | 62 | *no* | 19 Deletions in exon 19 | male |
| 301 | 56 | *no* | No mutation detected | female |
| 302 | 36 | *no* | No mutation detected | male |
| 303 | 66 | *yes* | No mutation detected | male |
| 304 | 58 | *yes* | No mutation detected | male |
| 305 | 73 | *no* | 19 Deletions in exon 19 | female |
| 306 | 55 | *yes* | No mutation detected | male |
| 307 | 57 | *yes* | No mutation detected | male |
| 308 | 49 | *yes* | No mutation detected | male |
| 309 | 65 | *no* | No mutation detected | male |
| 310 | 83 | *yes* | No mutation detected | male |
| 311 | 53 | *no* | 19 Deletions in exon 19 | male |
| 312 | 54 | *yes* | No mutation detected | male |
| 313 | 62 | *yes* | No mutation detected | male |
| 314 | 40 | *yes* | No mutation detected | male |
| 315 | 59 | *no* | No mutation detected | female |
| 316 | 65 | *yes* | No mutation detected | male |
| 317 | 69 | *no* | No mutation detected | male |
| 318 | 64 | *no* | No mutation detected | female |
| 319 | 52 | *no* | No mutation detected | female |
| 320 | 75 | *no* | 19 Deletions in exon 19 | male |
| 321 | 59 | *no* | L858R IN EXON 21 | male |
| 322 | 70 | *yes* | No mutation detected | male |
| 323 | 33 | *yes* | 19 Deletions in exon 19 | male |
| 324 | 70 | *yes* | No mutation detected | male |
| 325 | 54 | *no* | 19 Deletions in exon 19 | female |
| 326 | 74 | *yes* | L858R IN EXON 21 | male |
| 327 | 61 | *no* | 19 Deletions in exon 19 | female |
| 328 | 75 | *no* | 19 Deletions in exon 19 | male |
| 329 | 34 | *no* | T790M in exon 20 | female |
| 330 | 61 | *no* | No mutation detected | female |
| 331 | 54 | *no* | 19 Deletions in exon 19 | female |
| 332 | 60 | *yes* | No mutation detected | male |
| 333 | 58 | *yes* | No mutation detected | male |
